# SIRT3 protects against early brain injury following subarachnoid hemorrhage *via* promoting mitochondrial fusion in an AMPK dependent manner

**DOI:** 10.1186/s41016-019-0182-7

**Published:** 2020-01-03

**Authors:** Xun Wu, Jianing Luo, Haixiao Liu, Wenxing Cui, Dayun Feng, Yan Qu

**Affiliations:** Department of Neurosurgery, Tangdu Hospital, the Fourth Military Medical University, Xi’an, 710038 Shaanxi China

**Keywords:** Subarachnoid hemorrhage, SIRT3, Honokiol, Mitochondrial fusion, AMPK, Mfn1, Mfn2

## Abstract

**Background:**

Subarachnoid hemorrhage (SAH), an acute cerebrovascular accident, features with its high death and disability rate. Sirtuin3 (SIRT3) is a NAD+ dependent deacetylase which mainly located in mitochondria. Reduced SIRT3 function was indicated to involve in many disorders of central nervous system. Herein, we aimed to explore the neuroprotective effects of SIRT3 on SAH and to furtherly explore the underlying mechanisms.

**Methods:**

Adult C57BL/6 J male mice (8–10 weeks) were used to establish SAH models. The pharmacological agonist of SIRT3, Honokiol (HKL), was injected in an intraperitoneal manner (10 mg/kg) immediately after the operation. Brain edema and neurobehavioral score were assessed. Nissl staining and FJC staining were used to evaluate the extent of neuronal damage. The changes of mitochondria morphology were observed with transmission electron microscopy. Western blot was used for analyzing the protein level of SIRT3 and the downstream signaling molecules.

**Result:**

SIRT3 was downregulated after SAH, and additional treatment of SIRT3 agonist HKL alleviated brain edema and neurobehavioral deficits after SAH. Additionally, electron microscopy showed that HKL significantly alleviated the morphological damage of mitochondria induced by SAH. Further studies showed that HKL could increase the level of mitochondrial fusion protein Mfn1 and Mfn2, thus maintaining (mitochondrial morphology), protecting mitochondrial function and promoting neural survival. While, additional Compound C (CC) treatment, a selective AMPK inhibitor, abolished these protective effects.

**Conclusions:**

Activation of SIRT3 protects against SAH injury through improving mitochondrial fusion in an AMPK dependent manner.

## Background

Subarachnoid hemorrhage (SAH), an acute cerebrovascular accident, features with its high death and severe neurologic impairment [1, 2]. SAH can be induced by many events, including aneurysm rupture, hypertensive arteriosclerosis, severe trauma, arteriovenous malformation, or abnormal vascular network at skull base [3]. Recently, it was suggested that early brain injury (EBI) played a critical role in pathological process of SAH. EBI refers to the direct brain damage before the occurrence of delayed vasospasm. Growing evidences showed that EBI might contribute largely to mortality and morbidity at the early period 24–72 h after the SAH [4]. The mechanisms of EBI are complicated, which include inflammatory reaction, oxidative stress injury, brain edema, and glutamate excitotoxicity [5].

Mitochondrial dysfunction was recognized as a critical pathological factor in SAH injury. Mitochondria are highly dynamic and undergo fusion and fission all the time under both physiological stimulation and pathological injuries. Normal mitochondrial morphology is vital in the maintenance of mitochondrial function, energy metabolism, and calcium cycle and so on [6, 7]. Previous studies have confirmed that the damage of mitochondrial morphology contributed largely to the acute neurological injury [8, 9]. Mitofusins (Mfn1 and Mfn2), located onto outer membrane of mitochondria, play an indispensable role in regulating mitochondrial fusion. They participated in many cellular activities including oxidative stress, cell proliferation, cell apoptosis, and axonal transport of mitochondria [10–12]. However, the role of mitochondrial fusion in SAH has not been fully clarified.

Sirtuins are nicotinamide adenine dinucleotides (NAD)-dependent protein deacetylases [13]. SIRT3 is the most important deacetylase in mitochondria, which plays a central role in mitochondrial function and energy metabolism [14, 15]. SIRT3 was reported to protect against senile myocardial hypertrophy, tumors, aging-related diseases and so on [16, 17]. SIRT3 was also reported to be indispensable for the maintenance of mitochondrial dynamics in many diseases models. AMPK pathway participates in energy metabolism, helps to resist stress response, and promotes cell survival [18]. Previous studies have confirmed that there is a positive feedback loop between SIRT3and AMPK, which protected ischemic brain damage [19]. Honokiol (HKL) is natural compound with a small molecular weight and is extracted from the bark of Magnoliaceae. HKL was demonstrated to be able to directly bind to sirt3, −enhances both protein levels and enzymatic activity at the same time, thereby alleviating the damage induced by cardiac hypertrophy [20]. In this experiment, we explored whether the activation of SIRT3 protected against SAH and investigated the underlying mechanisms focusing on SIRT3/AMPK pathway and mitochondrial fusion.

## Methods

### Animals and ethics

These experiments were under the permission of the Ethics Committee of the Fourth Military Medical University and were strictly conducted according to the guidelines of the National Institutes of Health Guide for the Care and Use of Laboratory Animals. Healthy C57BL/6 J mice (weighed from 20 to 25 g, aged from 8 to 10 weeks, male) were purchased from the animal center of the Fourth Military Medical University. All mice were kept in animal facilities with specific pathogen-free conditions and fed well.

### SAH surgery and treatments

In vivo subarachnoid hemorrhage model was established as described as below. Anesthetized mice with 2% pentobarbital sodium and kept them in a suitable position. The skin was cut and the tissue was separated to uncover right common carotid artery bifurcation. Then after ligating and dissecting right external carotid artery, inserted the sharpened suture into the right internal carotid artery and pierced the artery approximately 10 to 12 mm inward from the common carotid artery bifurcation. And then insert 3 mm further for perforating the bifurcation of the anterior and middle cerebral arteries, staying about 20 s before withdrawal. Sham group mice were operated under the same experimental methods, except for the 3-mm insertion. HKL solution (10 mg/kg) was injected intraperitoneally immediately after the surgery.

### Experiment design

Experiment 1: To explore the changes of SIRT3 in various time points after SAH, 56 mice (8 mice came from Sham group, and 48 mice came from SAH group which were survived from the initial total 63 mice) were randomly and equally assigned into seven groups (each contained 8 mice): Sham group, and six experimental groups at various time points, including 3 h, 6 h, 12 h, 24 h, 48 h, and 72 h after SAH. All experimental mice were sacrificed in indicated time points following the surgery to detect SIRT3 level to choose a suitable time point for the following experiments.

Experiment 2: Exploring the underlying mechanism of the protective effects of SIRT3 in vivo. Ninety-six mice (24 mice came from Sham group and 72 mice are survived from the initial total 103 mice) were randomly and equally assigned into the groups listed as follows (each contained 24 mice): Sham group, SAH group, SAH + HKLgroup, and SAH + CC group 24 h following SAH; 24 mice were randomly divided three parts: 8 mice were tested for behavioral disturbances and then brain water content measurement; 8 mice were used for western blot and transmission electron microscopy; the last 8 mice were collected for Fluoro-Jade C (F-JC) staining and Nissl staining.

### Measurement of brain water content

Brain edema was assessed at 24 h after SAH. Mice brain was weighed once after the removal to record the wet weight, then put it in the oven with temperature from 95 to 100 °C for 72 h to get the dry weight. Brain water content was assessed and calculated through a computational formula: (wet weight -dry weight)/wet weight × 100%.

### Modified neurological severity score

We take out the modified neurological severity score (mNSS) to assess the extent of neurological functional impairments 24 h after SAH as previous study described [21]. The mNSS which contains sensory, motor, and reflex tests was graded from 0 score to18 score (0 score refers to the normal score; 18 score refers to the maximal deficit score). These experiments were conducted by two observers who were blind to all groups.

### Nissl staining

After anesthesia, mice were perfused with pentobarbital sodium; mice brains were removed and fixed with paraformaldehyde for approximately 4 h, following by being dehydrated in doses of 10%, 20%, and 30% sucrose in turn. Then brain tissue was embedded in paraffin and sliced into sections about 15 μm. Then, dewaxed and rehydrated the slices. Stained selected brain slices with 1% methyl violet (10–20 min), then treated with Nissl differentiation solution (4–8 s) and rinsed with purified water. The slices were then treated with gradient ethanol and xylene. Finally, observed and photographed the sections under an optical microscope.

### FJC staining

The brains were removed, fixed, dehydrated, and sliced into pieces about 15 μm as described above. The slices were then incubated with 1% sodium hydroxide solution in 80% ethanol (5 min), then rehydrated in 70% ethanol and purified water (2 min) respectively. Slices were incubated with 0.06% potassium permanganate (10 min), washed with purified water (3 min), and incubated with 0.0001% F-JC solution (Millipore, Temecula, CA, USA 15 min). The slices were then washed three times with purified water for 1 min each time. The images were collected by A1 Si confocal microscope (Nikon, Japan), calculating the number of FJC positive neurons.

### Transmission electron microscope

Mice were sacrificed and perfused at 24 h after SAH operation as described above, which was followed by perfusion of 0.9% saline and 4% paraformaldehyde, respectively. Mice were sacrificed and perfused at 24 h after SAH operation as described above. Brains were removed and tissues around hematoma were cut and trimmed into 1–2 mm wide blocks which were then fixed in 4% glutaraldehyde for overnight. The slices were then post-fixed with 1% osmium tetroxide (1 h). After that, they were dehydrated with ethanol immersion, which was followed by embedded in resin. Then, cut the pieces into 80-nm sections. Lastly, observed the ultrathin sections with a JEM-1400 electron microscope (JEOL, Tokyo, Japan) and captured the micrographs with a charge coupled device camera (Olympus, Tokyo, Japan).

### Western blot

We separated protein samples with SDS-PAGE gels. After that, it was transferred to PVDF membranes (Millipore, USA). Then blocked the membranes with non-fat milk (5%) which was diluted with TBST, and incubated the membranes with primary antibodies overnight at 4 °C. Second day, after three times 10-min TBST washes, we incubated the membranes with the appropriate secondary antibodies (1:5000) at 27 °C for 2 h. Lastly, after the three times of 5-min TBST washes, detect the protein bands with a BioRad imaging system (Bio-Rad, USA). The primary antibodies were listed as below: SIRT3(1:1000, C73E3, Cell Signaling), Ac-lys (1:1000,9441S, Cell Signaling), phospho-AMPK (1:1000, D4D6D, Cell Signaling), AMPK (1:1000, 2532S, Cell Signaling), Mfn2 (1:1000, D2D10, Cell Signaling), Mfn1 (1:1000, A9880, ABclonal), Bax (1:1000, gtx32465, Gene Tex), Bcl2 (1:1000, gtx100064, Gene Tex),β-actin(1:3000, wh096194, Wanleibio).

### Statistical processing

All data were presented as mean ± SD. GraphPad Prism 6.0 software (GraphPad, USA) was used for the following statistical analysis. Comparison between multiple groups used one-way analysis of variance (ANOVA), following with the Tukey post hoc test for the analysis. Comparison between two groups used Student’s *t* test (unpaired, two-tailed) for the analysis. Differences with *P* values < 0.05 were identified as statistically significant.

## Results

### SIRT3 expression after SAH injury in mice

**Fig. 1:**
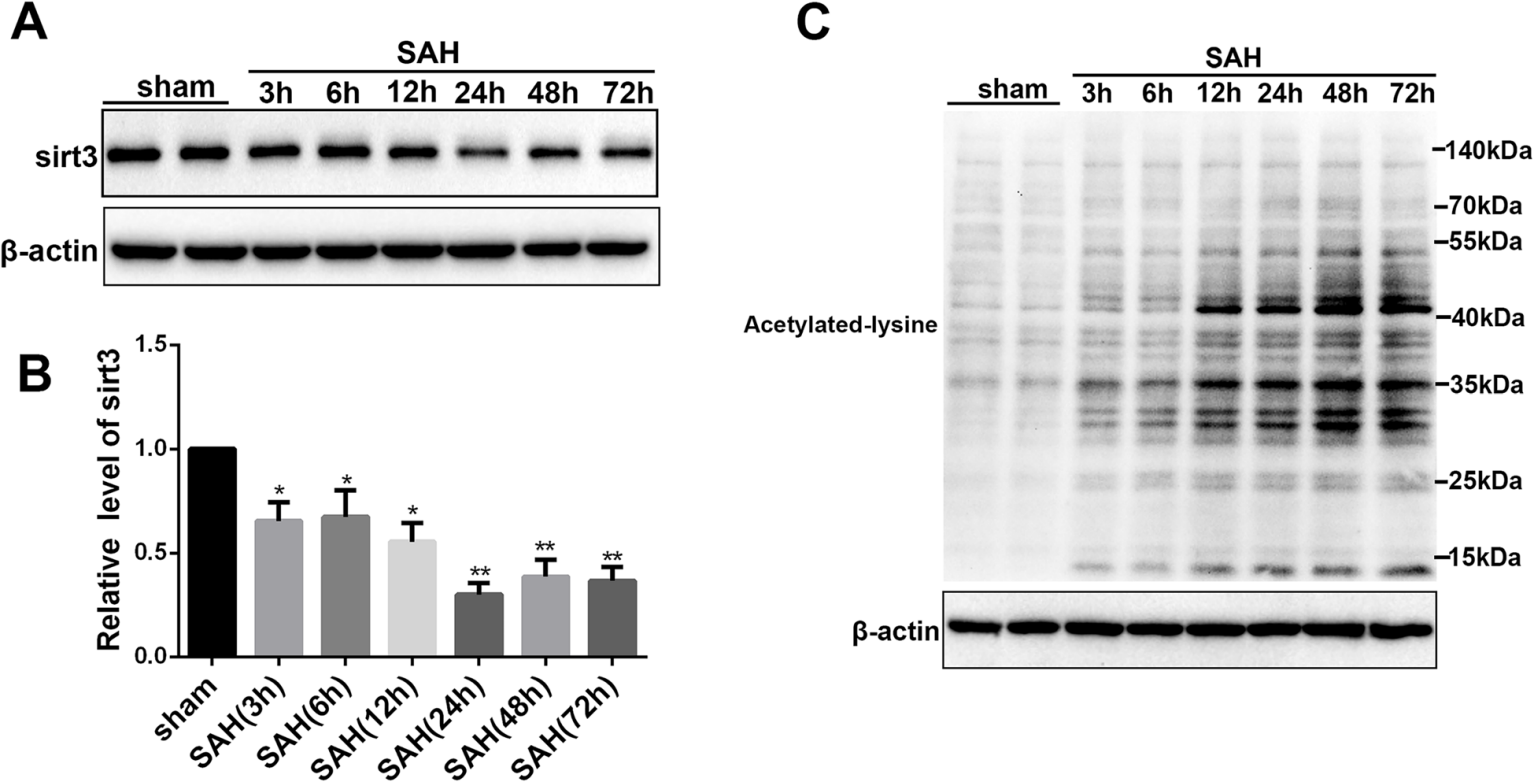
SIRT3 expression after SAH in mice. **a** Western blot analysis of the protein level of SIRT3 after SAH. **b** The bar graph shows the statistical results of SIRT3 protein level. **c** Western blot analysis of the acetylation level of mitochondrial proteins. Values were represented as mean ± SD, *n* = 8 for each group. *P < 0.05 and **P < 0.01 vs. sham group

In order to explore the time course of SIRT3-level in SAH, we detected the SIRT3 protein level at different time points including 0 (sham group), 3, 6, 12, 24, 48, and 72 h after SAH. The experiment revealed that the protein level of SIRT3 declined to the minimum at 24 h following SAH (Fig. 1a, b *P* < 0.05). SIRT3 is located in mitochondrial matrix and it plays a central role in regulating mitochondrial function via protein deacetylation to enhance their enzymatic activity. Disruption of SIRT3 activity contributed to the increased acetylation of mitochondrial proteins and impaired mitochondrial function. Then, we isolated the mitochondria to detect the acetylation level of mitochondrial proteins. Compared with sham group, the level of acetylation in SAH group was increased in a time-dependent manner (Fig. 1c, *P* < 0.05). These showed that the level of SIRT3 was decreased and the function of SIRT3 was impaired after SAH. So we take 24 h after SAH as the time point for the following experiments.

### SIRT3 agonist HKL alleviated neurological function deficits and brain edema after SAH in mice

**Fig. 2:**
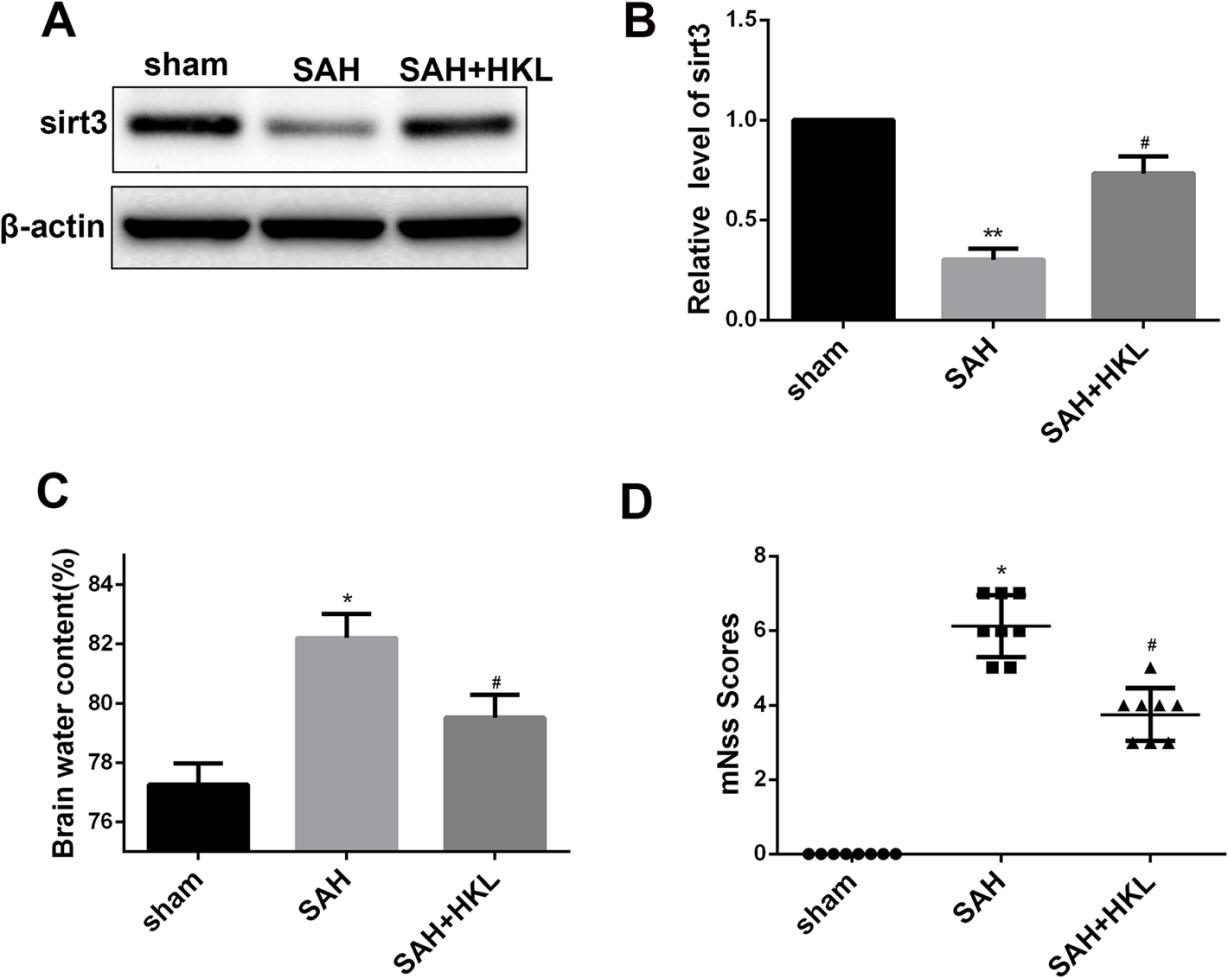
SIRT3 agonist HKL alleviated neurological deficits and brain edema after SAH in mice. **a**, **b** HKL treatment significantly reversed the low expression of SIRT3 induced by SAH injury. **c** The modified neurological severity scores in each group. **d** Brain water content in each group. Values are represented as mean ± SD. *n* = 8 for each group. *P < 0.05 and **P < 0.01 vs. sham group, ^#^*P* < 0.05 and ^##^*P* < 0.01 vs. SAH group

Western blot indicated that additional HKL treatment significantly reversed the low level of SIRT3 induced by SAH injury (Fig. 2a, b *P* <  0.05). Then we measured brain water content and took behavioral tests of mice to evaluate the effect of HKL on SAH. The results revealed that brain water content increased significantly after SAH injury, which were significantly reduced by additional HKL treatment (Fig. 2c, *P* < 0.05). Similarly, we found significant neurological impairment emerged after SAH injury, while HKL treatment significantly improved the neurological function score after SAH (Fig. 2d, *P* < 0.05).

### HKL ameliorated neuronal damage after SAH

**Fig. 3:**
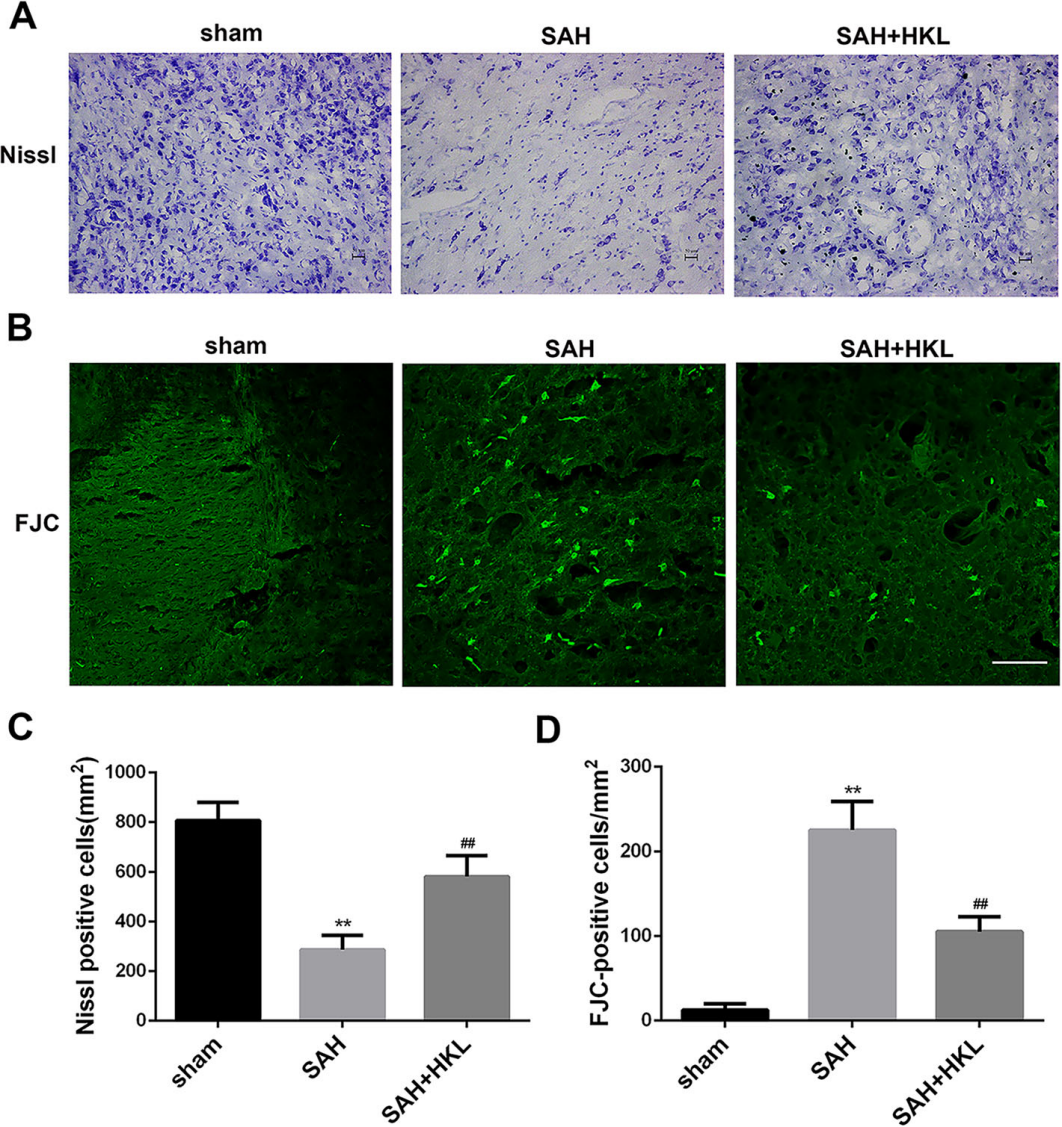
HKL ameliorated neuronal damage after SAH in mice. **a** Representative images of Nissl staining and **c** quantitative analyses of Nissl positive neurons in each group 24 h after the operation are shown. Scale bar 50 μm. **b** Representative images of FJC staining and **d** Quantitative analyses of FJC-positive cells in each group 24 h after the operation are shown. Scale bar 50 μm. Values were represented as mean ± SD, *n* = 8 for each group. *P < 0.05 and **P < 0.01 vs. sham group, ^#^*P* < 0.05 and ^##^*P* < 0.01 vs. SAH group

Nissl staining and FJC staining were used to assess the effects of HKL on neural damage after SAH. Normal neurons feature with cytoplasmic Nissl staining. In contrast, loss of Nissl staining suggests the damaged neurons. The extent of neural loss was evaluated with the decreased number of Nissl-positive cells (Fig. 3a, c). The number of Nissl-positive cells was observed to be significantly downregulated in the SAH group (vs. sham group, *P* < 0.05), which was significantly reversed after the treatment with HKL (vs. SAH group, *P* < 0.05). In addition, the protective effects were furtherly validated by FJC staining. FJC-positive cells represent degenerated neurons. We found that SAH insignificantly increased FJC-positive cells, which were reversed by additional HKL treatment (Fig. 3b, d, *P* < 0.05), indicating that HKL could alleviate neuronal damage after SAH.

### HKL alleviated morphological damage of mitochondria after SAH

**Fig. 4:**
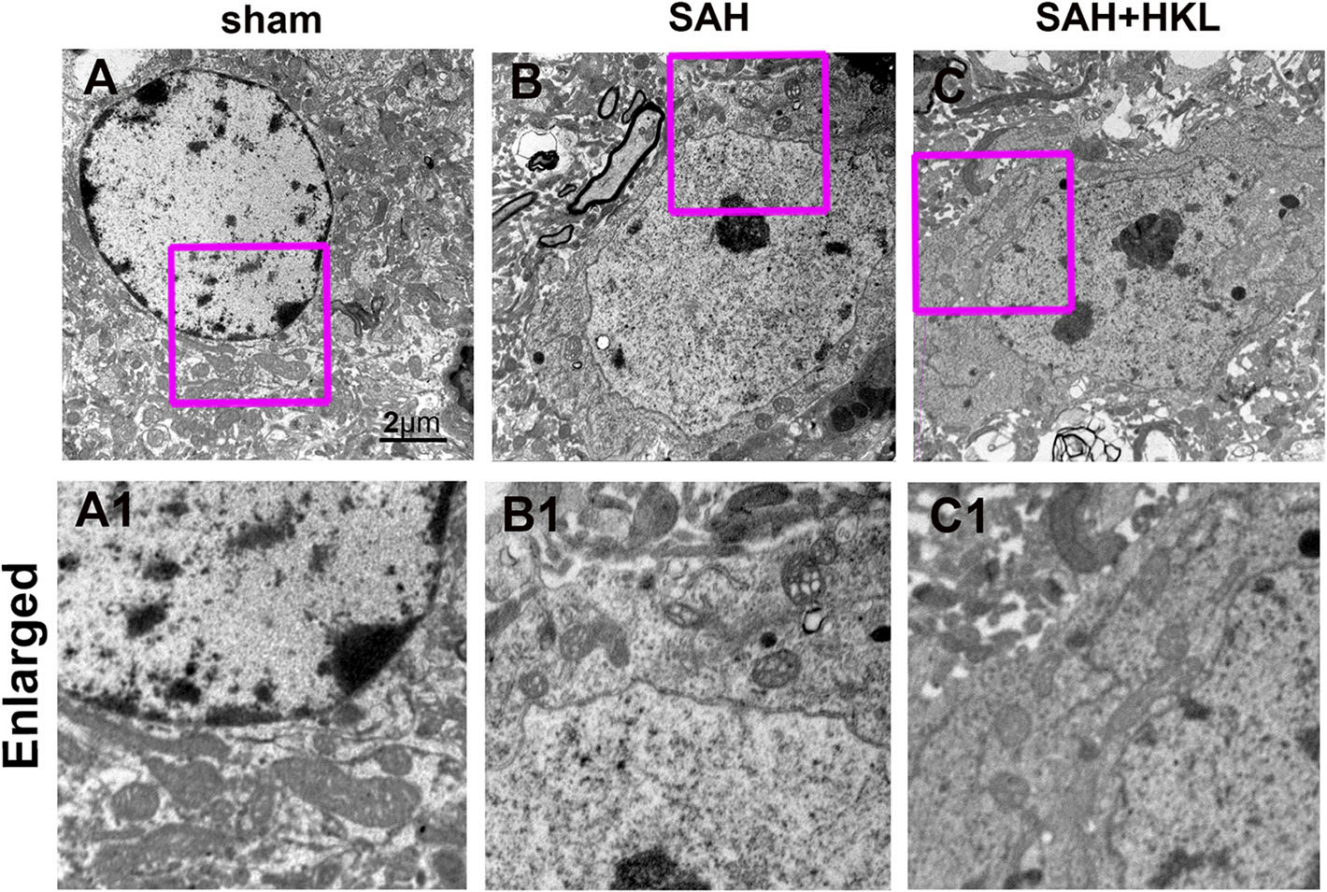
Effects of HKL on the ultrastructure of neurons 24 h after SAH, as detected using electron microscopy. **a**–**c** Representative ultrastructure of neurons is shown in each group. **a**1–**c**1 Representative ultrastructure of neurons are enlargements of **a**–**c** respectively. Scale bar = 2 μm

Electron microscopy was used to study the changes of mitochondrial morphology and structure. In the sham group, neurons were featured by long tubular mitochondrial structures, apparent cristae appearance, and some small globular mitochondrial structures (Fig. 4a, a1. Mitochondrial abnormalities were observed in neurons after SAH. In SAH group, amounts of small globular mitochondrial structures with mitochondrial fragments were observed. In addition, some mitochondria showed abnormal morphology after SAH, including swelling mitochondrial, ridge collapse, and membrane rupture (Figs. 4b, b1). Importantly, HKL therapy increased mitochondrial tubular networks, significantly reduced mitochondrial fragments, and largely maintained normal mitochondrial morphology (Fig. 4c, c1).

### SIRT3 promoted mitochondrial fusion after SAH in an AMPK dependent manner

**Fig. 5:**
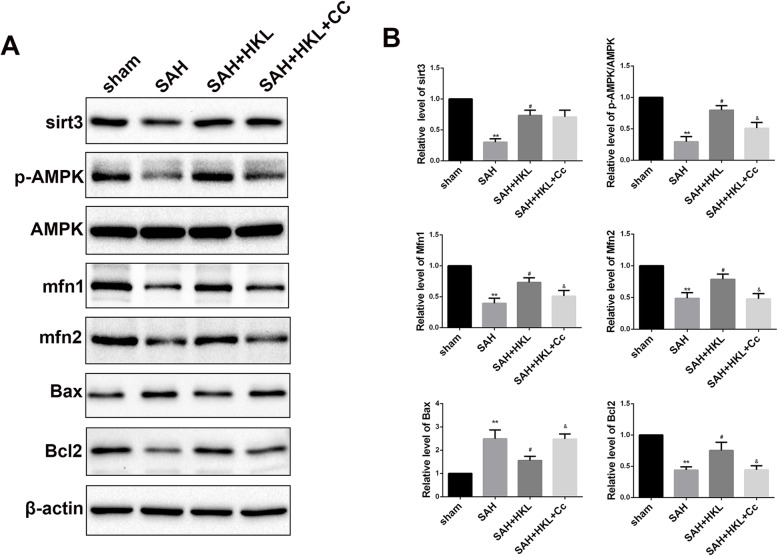
SIRT3 protected mitochondrial fusion proteins Mfn1 and Mfn2 after SAH in an AMPK-dependent manner. (a selective inhibitor of AMPK). **a** Western blotting analysis of SIRT3; p-AMPK; AMPK; Mfn1, Mfn2; Bcl2; Bax in each group. **b** The bar graph shows the statistical results of protein level. CC: Compound C. Values were represented as mean ± SD, *n* = 8 for each group. *P < 0.05 and **P < 0.01 vs. sham group, ^#^*P* < 0.05 and ^##^*P* < 0.01 vs. SAH group. ^&^*P* < 0.05 vs. SAH + HKL group

Western blotting results showed that the expressions of SIRT3, p-AMPK/AMPK, Mfn1, Mfn2, and Bcl2/Bax in SAH group were significantly declined than those in sham group (Fig. 5a, b, *P*<0.05). However, the expression of SIRT3, p-AMPK/AMPK, Mfn1, Mfn2, and Bcl2/Bax significantly increased after HKL administration (Fig. 5a,b, *P*<0.05), suggesting that HKL activated the SIRT3 and AMPK pathway, upregulated the expression of Mfn1 and Mfn2, protected mitochondrial morphology, and promoted neuron survival. CC treatment, a selective inhibitor of AMPK pathway, can largely counteract the protective effect of HKL (Fig. 5a, b, *P*<0.05). It suggested that the protective effects of SIRT3 activator HKL on mitochondrial morphology were depended on the AMPK pathway.

## Discussion

SAH is a life-threatening cerebrovascular emergency and features with high mortality and disability rates. Because the pathological process of SAH is complicated, the efficient intervention is still lacking. Mitochondrial dysfunction is identified as a major contributor to the early brain injury of SAH [22, 23].Herein, we explored the role of SIRT3, which is a NAD^+^-dependent deacetylase located in mitochondria, in SAH-induced brain injury. Previous researches indicated that SIRT3 protected against brain injury by inhibiting the activation of HIF1a and ROS-mediated oxidative damage [24]. In this experiment, we found the protein level and deacetylation function of SIRT3 were significantly decreased after SAH. It is suggested that the impairment of SIRT3 may be one of the major factors contributing to the EBI of SAH. Then, after HKL treatment, brain edema, neurological dysfunction, and neuronal damage induced by SAH were significantly alleviated. It indicated that activation of SIRT3 could effectively protect SAH-induced brain injury.

Our further studies found that HKL significantly attenuated the morphological damage of mitochondria. Mitochondria constantly undergo fission and fusion, whose balance is indispensable for the maintenance of normal mitochondrial function. Mitofusins (Mfn1 and Mfn2) are of vital importance in regulating mitochondrial fusion. Previous studies showed that damaged mitochondrial oxidative phosphorylation and declined ATP production happened when Mfn2 was deleted [11, 12]. The mitofusins protein also—seemed to play additional roles in many process besides mitochondrial fusion. Mfn2 was found to take part in the axonal transport of mitochondria [25] and also was indicated in tethering endoplasmic reticulum (ER) and mitochondria [26]. Herein, we found HKL treatment can significantly elevate the level of Mfn1 and Mfn2, which may contribute the normalization of normal mitochondrial fusion.

AMPK pathway was demonstrated to play a critical role in regulating cellular energy metabolism, such as mitochondrial biogenesis, adaptive thermogenesis, glucose/fatty acid metabolism [18]. Previous studies implicated that there is a positive feedback loop between SIRT3and AMPK [19]. Meanwhile, the activation of AMPK pathway was reported to promote the expression of Mnf1 and Mfn2 [8, 27]. In the current study, we found CC treatment, which is a selective inhibitor of AMPK pathway, counteracted the protective effect of HKL on mitochondrial fusion to a large extent. All these suggested that the protective effects of HKL on mitochondrial morphology relies on the activation of SIRT3/AMPK pathway.

There are several limitations have to be pointed out in this study. Firstly, we mainly discuss the protective effects of HKL and SIRT3 on EBI after SAH. Future studies should further evaluate the long-term effect. Secondly, this study only focuses on the role of SIRT3 in regulating mitochondria fusion. We should also investigate the interaction of mitochondria with many organelles, such as endoplasmic reticulum, in the maintenance of normal physiological activity next time. Thirdly, although we have achieved satisfactory results in animal experiments, more studies including larger samples and more advanced technology applications are needed for the future clinical application.

## Conclusion

Our study indicated that the activation of SIRT3 protected against SAH injury, and the underlying mechanism may be related to the regulation of mitochondrial fusion in an AMPK dependent manner.

## Data Availability

The datasets used and/or analyzed during the current study are available from the corresponding author on reasonable request.

## Abbreviations

AMPK: Adenosine 5′-monophosphate (AMP)-activated protein kinase
F-JC: Fluoro-Jade C
HKL: Honokiol
mNSS: Modified neurological severity score
SAH: Subarachnoid hemorrhage
SIRT3: Sirtuin3
